# Red Light Mitigates the Deteriorating Placental Extracellular Matrix in Late Onset of Preeclampsia and Improves the Trophoblast Behavior

**DOI:** 10.1155/2022/3922368

**Published:** 2022-04-20

**Authors:** Jakara Griffin, John G. Krolikowski, Kenisha Kounga, Janine Struve, Agnes Keszler, Brian Lindemer, Michelle Bordas, Grant Broeckel, Nicole L. Lohr, Dorothee Weihrauch

**Affiliations:** ^1^Department of Anesthesiology, Medical College of Wisconsin, 8701 Watertown Plank Road, Milwaukee, WI 53226, USA; ^2^Department of Orthopedic Surgery, Medical College of Wisconsin, 8701 Watertown Plank Road, Milwaukee, WI 53226, USA; ^3^Department of Medicine, Medical College of Wisconsin, 8701 Watertown Plank Road, Milwaukee, WI 53226, USA

## Abstract

Preeclampsia is a serious pregnancy disorder which in extreme cases may lead to maternal and fetal injury or death. Preexisting conditions which increase oxidative stress, e.g., hypertension and diabetes, increase the mother's risk to develop preeclampsia. Previously, we established that when the extracellular matrix is exposed to oxidative stress, trophoblast function is impaired, and this may lead to improper placentation. We investigated how the oxidative ECM present in preeclampsia alters the behavior of first trimester extravillous trophoblasts. We demonstrate elevated levels of advanced glycation end products (AGE) and lipid oxidation end product 4-hydroxynonenal in preeclamptic ECM (28%, and 32% increase vs control, respectively) accompanied with 35% and 82% more 3-chlorotyrosine and 3-nitrotyrosine vs control, respectively. Furthermore, we hypothesized that 670 nm phototherapy, which has antioxidant properties, reverses the observed trophoblast dysfunction as depicted in the improved migration and reduction in apoptosis. Since NO is critical for placentation, we examined eNOS activity in preeclamptic placentas compared to healthy ones and found no differences; however, 670 nm light treatment triggered enhanced NO availability presumably by using alternative NO sources. Light exposure decreased apoptosis and restored trophoblast migration to levels in trophoblasts cultured on preeclamptic ECM. Moreover, 670 nm irradiation restored expression of Transforming Growth Factor (TGF*β*) and Placental Growth Factor (PLGF) to levels observed in trophoblasts cultured on healthy placental ECM. We conclude the application of 670 nm light can successfully mitigate the damaged placental microenvironment of late onset preeclampsia as depicted by the restored trophoblast behavior.

## 1. Introduction

Preeclampsia (pre-E) is a disorder characterized by the onset of hypertension and proteinuria identified after 20 weeks of gestation. There is a wide spectrum of sequelae identified in pre-E, including liver dysfunction, kidney failure and in extreme circumstances seizures and death. Placental integrity is a key aspect of healthy pregnancy; therefore, a healthy cellular microenvironment is paramount for correct placentation. Extravillous trophoblasts migrate into uterine arteries, embed and remodel the vasculature so as to appropriately increase placental blood flow o, and provide the fetus with oxygen and nutrients. Increased oxidative stress is one of the causes of pre-E [1], and oxidatively altered matrix is a hallmark of the altered cellular microenvironment which propagates disease development through dysregulated placentation. Previously, we demonstrated that oxidative stress alters the extracellular matrix (ECM) which leads to an oxidized cellular microenvironment. Oxidative adducts on ECM (e.g., advanced glycation end products (AGE) and the lipid peroxidation end product 4-hydroxynoneal) are well described in several disease states like scleroderma and diabetes but have not been characterized in pre-E [2, 3].

The oxidative environment described in pre-E disrupts the balance between vascular maintenance factors such as nitric oxide (NO) and vascular inhibitors such as superoxide t. NO bioavailability in pre-E is reduced [4, 5]. Here, we hypothesized that red light treatment can reverse the deleterious effects of oxidative stress on the ECM and maintain healthy trophoblast population by increasing bioavailable NO. Photobiomodulation is a treatment modality using red/near infrared light, and application of 670 nm wavelength specifically improves the cellular microenvironment and restores cell behavior by increasing NO bioavailability [2, 6]. The focus here is on the consequences of increased oxidative stress on placentas in late onset pre-E (after 34 weeks of gestation) and how these changes influence first trimester trophoblast behavior and first trimester trophoblast signaling. We studied how oxidation impacts the ECM of decellularized placentas. Moreover, first trimester extravillous trophoblasts were cultured on the ECM from healthy and preeclamptic placentas. We found that the microenvironment changed by oxidation influenced the behavior of first trimester extravillous trophoblasts cultured on placental ECM (pECM). As a treatment, photobiomodulation with 670 nm light reverses the changes induced by oxidative stress.

## 2. Methods

### 2.1. Human Samples

Healthy and preeclamptic human placental samples (4 healthy and 4 preeclamptic) collected upon delivery were obtained from the tissue bank (IRB#00029926). Samples were used for histology on frozen sections, isolation of total protein and samples were decellularized for in vitro studies.

### 2.2. Isolation of Placental Extracellular Matrix

The placental samples were put into a beaker, and 15 mL hypertonic 1% sodium dodecyl sulfate (SDS) was added. The samples were agitated in 1% SDS solution for 18 h at room temperature. The decellularized placental samples were washed for 30 min in 15 mL 0.5% Triton X-100 followed by a wash for 15 min with 15 mL PBS. The extracellular matrix was snap frozen in liquid nitrogen and powderized with a precooled mortar and pestle. A suspension of the pulverized matrix was prepared in PBS containing 1% antibiotic (typically 300 *μ*L) and sonicated for 30-60 s on the high setting on ice. The protein concentration is determined using a Bradford assay.

### 2.3. 670 nm Light Treatment

100 mW/cm^2^ LED light source (NIR Technologies, Waukesha, WI) was applied once for 40 seconds directly to the slides. Power output was determined with X97 Optometer (Gigahertz Optic Gbmh, Turkenfeld, Germany). Photobiomodulation was applied on extravillous trophoblasts cultured on pECM.

### 2.4. Extravillous Trophoblast Cell Culture

HTR8/SVneo first trimester extravillous trophoblasts were used as a surrogate. They were purchased from ATCC (ATCC® CRL-3271™, Manassas, VA) and cultured with RPMI 1640 media containing 5% fetal bovine serum (GIBCO, ThermoFisher Scientific, Carlsbad, CA). 10,000 cells per well were seeded on pECM derived from healthy and preeclamptic patients coated 4-well chamber slides or 100 mm cell culture dishes. pECM was used in a concentration of 20 *μ*g/mL. HTR8/SVneo cells were cultured for 24 hours on the different ECM.

### 2.5. Nitric Oxide Levels

To determine the nitric oxide levels, HTR8/SVneo was cultured on the various matrices. HTR8/SVneo was incubated for 30 min with 4-amino-5-methylamino-2′7′diaminofluorescein (DAF; 10 *μ*M; Invitrogen, Carlsbad, CA) for the determination of nitric oxide (NO) levels. The fluorescent stain was removed, and the cells were allowed to stabilize for 30 min. The slides were then sealed with an aqueous mounting media and analyzed immediately by confocal microscopy (Eclipse TE2000-U; Nikon). Images were recorded with EZ-C1 2.10 software (Nikon). Images were captured using 488 nm excitation for and >519 nm emission, respectively. Fluorescence intensity was measured by determining pixel intensity of each cell, and only viable cells were counted.

### 2.6. Immunohistochemistry

To visualize changes in the extracellular matrix, 15 *μ*m frozen sections from healthy and preeclamptic placentas were cut and adhered to slides. The sections were fixed in 4% paraformaldehyde in PBS and permeabilized in 1% Triton X in PBS. Primary antibodies for heparan sulfate proteoglycan 2 (Boster Biological Technology, Pleasanton CA, USA, Catalog # PB9277), collagen-1 (cat# 600-401-103, Rockland Immunochemicals, Limerick, PA), and galectin-3 (cat#sc-32790, Santa Cruz Biotechnologies, Santa Cruz, CA), as well as PLGF (1: 50, cat#MBS713706, MyBioSource,,San Diego CA) and TGF*β* (cat#NBP2-22114, 1 : 500 dilution, Novus Biologicals, Centennial, CO) were applied and incubated for 30 minutes at 37°C. All three proteins are not only structurally important but are also involved in signaling processes. After 3 times 5-minute wash steps with PBS, the secondary antibodies (Alexa 488 conjugated, Invitrogen, Carlsbad, CA) were applied for 30 minutes at 37°C followed by three-times 5-minute wash. The slides were mounted with an aqueous mounting media. Additionally, HTR8/SVneo cells cultured on the two different placental ECM were treated with 670 nm light for 2 minutes at 50 mW. After 670 nm light treatment, the cells were immediately fixed with 4% paraformaldehyde followed by 1% Triton X for permeabilization. After a one step 5-minute PBS wash, the primary antibodies for PLGF (1 : 100 dilution, cat# CAU28581, Biomatik, Wilmington, DE) and TGF *β* (1 : 50 dilution, cat# sc-130348, Santa Cruz Biotechnologies, Santa Cruz, CA) were applied for 30 minutes at 37°C. The secondary Alexa 488 antibodies (Invitrogen, Carlsbad, CA) were chosen accordingly and incubated at 37°C for 30 minutes followed by two times 5-minute wash. The third wash step contained the nuclear stain DAPI.

### 2.7. Determination of sFlt-1 Levels by ELISA

To determine the by trophoblasts released sFlt-1 levels, sFlt-1 was measured by ELISA. The media from trophoblasts cultured on healthy and preeclamptic pECM were collected after 3 days, and an ELISA was performed according to manufacturer's instructions (cat# BMS268-3, Invitrogen, Waltham, MA).

### 2.8. Western Blot Analysis

To identify oxidized adducts, western blot analyses were performed. The focus was on advanced glycation end products (AGE, 1 : 500, cat#MBS540613, My Biosource, San Diego, CA), 4-hydroxynonenal (4HNE, 1 : 1000 dilution. Cat#MAB3249, R&D Systems, Minneapolis, MN), 3-chlorotyrosine (3-Cl-Tyr, 1 : 500 dilution, 3-ClTcat# HP5002, cell sciences, Canton MA), and 3-nitrotyrosine (3-NTyr, 1 : 200 dilution, cat# sc-32757, Santa Cruz Biotechnologies, Santa Cruz, CA). To gain insight into the interaction of endothelial nitric oxide synthase (eNOS, 1 : 200 dilution, cat# ab5589, Santa Cruz Biotechnologies, Santa Cruz, CA), phosphorylated eNOS Ser1177 (1 : 1000 dilution, cat#9570S, Cell Signaling, Danvers, MA) and heat shock protein 90 (Hsp90, 1 : 200 dilution, cat#sc-13119, Santa Cruz Biotechnologies, Santa Cruz, CA), a chaperone for eNOS, expression levels in placentas were assessed on total protein from healthy and pre-E placental samples. Densities of the bands were measured in arbitrary units with the BioRad ChemiDoc (Biorad, Hercules, Ca, GE Healthcare, Piscataway, NJ, USA). Briefly, total protein from homogenated placentas and from decellularized placentas were separated on a Sodium Dodecyl Sulfate Polyacrylamide Gel Electrophoresis gel. Equal protein loading (30 *μ*g/well of total protein) was ensured by *β*-actin loading control. Nitrocellulose membranes were used to visualize the proteins. The membrane was blocked with blocking buffer (cat# 37570, Protein-Free Blocking Buffer, Thermo Scientific Pierce) for 1 hour followed by 5 times 5-minute washes with Tris buffer saline. The primary antibodies were incubated at 4°C overnight. The next day, the blot was washed five times for 5-minute with TBS containing 1% tween, and the secondary antibodies were applied to incubate for 1 hour at room temperature. Bands were visualized with chemiluminescent SuperSignal™ West Pico PLUS Chemiluminescent Substrate (cat#34579, ThermoFisher, Waltham, MA) with the BioRad ChemiDoc (Biorad, Hercules, Ca, GE Healthcare, Piscataway, NJ, USA) [2].

### 2.9. Immunoprecipitation Assay

To determine the association between eNOS and phospho-eNOS and eNOS and Hsp90, immunoprecipitation of these proteins was performed with the same antibodies used in the western blot analyses, and then, the immunoprecipitates were blotted for phospho-eNOS and Hsp90, respectively. Briefly, the protein homogenate was precleared for 20 minutes with protein A agarose beads, followed by an antibody incubation overnight to bind the protein of interest to be pulled down. Protein A beads were then added, and based on increased affinity, protein A beads bound to the antibody. After 2 hours of incubation, the beads were pelleted by centrifugation, the complex separated from the beads by 95°C for 5 minutes, and the beads were removed. Western blots were performed as described above for each protein of interest (*n* = 4) [2].

### 2.10. Proliferation and Migration

Human first trimester extravillous trophoblast cell line HTR8/SVneo (ATCC, CRL-3271) was cultured on placental matrices (20 *μ*g/mL) in 24-well plates. HTR8//SVneo cells were cultured for 72 hours. HTR8/SVneo proliferation was determined by hemocytometer.

For migration studies, HTR8/SVneo cells were cultured on fibrin gel mixed with placental ECM from healthy and preeclamptic patients. Plasminogen-free human fibrinogen (5 mg/mL; cat# 341578, Millipore, Burlington, MA) was dissolved in serum-free medium, filtered through 0.2 *μ*m filters, and 20 *μ*g/mL ECM was added. Fibrin matrices were prepared by polymerization using thrombin (2.5 U/mL, Sigma, Saint Louis, MO, 2 h at 37°C). After polymerization, thrombin was inactivated using culture medium containing 10% fetal bovine serum (FBS) for 2 h at 37°C. HTR8/SVneo was seeded on to 24-well plates for 5 days. Images (200x) were captured, and an electronic grid was superimposed on each image. The number of tubes intersecting the squares was counted using Nikon Element software.

### 2.11. Apoptosis Assay

HTR8/SVneo cells were cultured on healthy and preeclamptic placental ECM in 4 chamber slides. Twenty-four hours later, a TUNEL assay was performed using an ApopTag Plus Fluorescein-labeled kit (cat# S7100, Millipore, Burlington, MA) per manufacturer's instructions. Briefly, 3′OH-DNA termini were labeled in situ with a fluorescein conjugated antidigoxigenin antibody. Slides were mounted and analyzed by confocal microscopy (Nikon Eclipse TE2000, EZ-C1-2.10 software, Nikon, Melville NY). Images were captured using 488 nm excitation for fluorescein. Green fluorescing cells were counted using Nikon Element software.

## 3. Results

### 3.1. Soluble Flt Release Was Increased Contributing to the Development of Late Onset Preeclampsia

**Figure 1:**
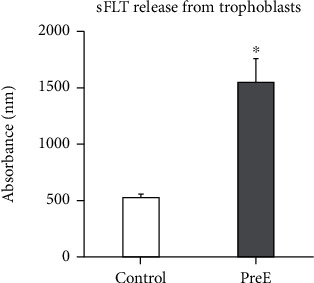
Soluble Flt release was increased in late onset preeclampsia (*p* ≤ 0.05, *n* = 4).

sFlt, a truncated version of VEGFR1, is a putative pre-E biomarker because its release is increased in pre-E [7, 8]. We characterized pre-E by measuring sFlt levels with ELISA (Figure 1). HTR8/SVneo released more sFlt when cultured on pECM from patients diagnosed with preeclampsia (pre-E: 1549 ± 207.85) compared to HTR8/SVneo cells cultured on pECM from healthy patients (control: 519 ± 33.2).

### 3.2. The Cellular Milieu of pECM in Late Onset Pre-E Is Changed

**Figure 2:**
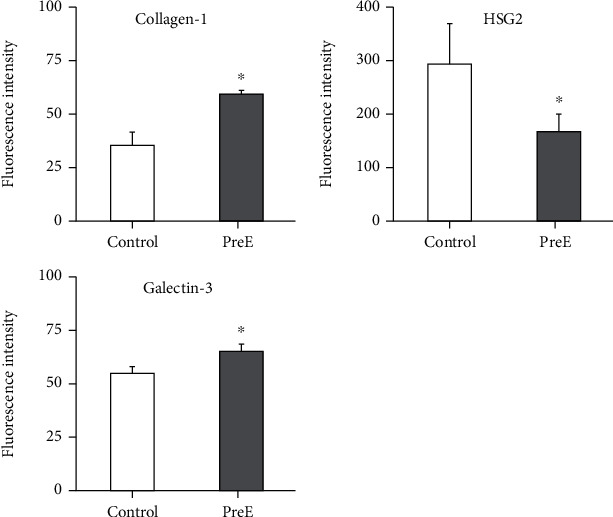
Critical components of the pECM were analyzed to establish changes in their expression. Collagen-1 expression is significantly increased, heparan-sulfate proteoglycan 2 is significantly decreased, and galectin-3 is significantly increased in pECM.

Critical components of the pECM were analyzed to establish changes in their expression (Figure 2). Collagen-I expression was upregulated in pECM from preeclamptic patients compared to pECM from healthy patients (control: 35.49 ± 6; pre-E: 58.85 ± 2.09). Heparan sulfate proteoglycan 2 expression was downregulated in the pECM from preeclamptic patients when compared with the pECM from healthy patients (control: 290.92 ± 86.6; pre-E: 169.42 ± 40.21). Galectin-3 expression is downregulated in the pECM of patients suffering from pre-E when compared to the pECM from healthy patients (control: 54.29 ± 2.48; pre-E: 66.76 ± 3.23).

### 3.3. eNOS, HSP90, and Phospho-eNOS Expression Levels Are Not Changed in Late Onset Pre-E

**Figure 3:**
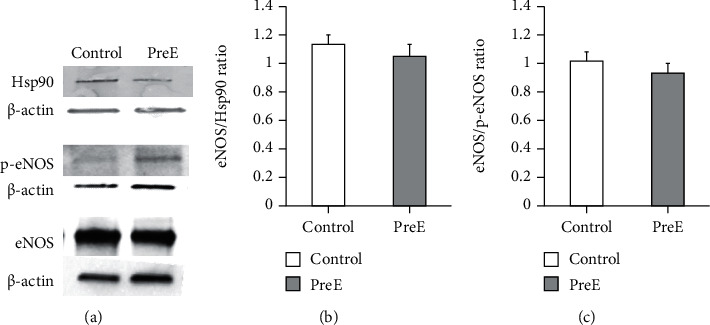
(a) eNOS, HSP90, and phospho-eNOS expression levels are not significantly changed in late onset pre-E. (b) Ratios of eNOS to phospho-eNOS and (c) eNOS to Hsp90 are not changed in late onset pre-E (*p* ≤ 0.05, *n* = 4).

NO is a key modulator in placentation [9, 10]. The coupling of phosphorylated eNOS to its chaperone HSP90 is of great importance to the synthesis of NO. If eNOS is uncoupled from HSP90, the enzyme switches to superoxide synthesis [11] resulting in decreased NO levels and a potential production of peroxynitrite oxidant. Therefore, we measured eNOS, HSP90, and phospho-eNOS expression levels with western blot and normalized them to *β*-actin. The expression levels were unchanged comparing control and late onset preeclampsia sample (Figure 3(a)). The values are as follows: eNOS: control 0.51 ± 0.06, pre-E 0.507 ± 0.086; HSP90: control 1.02 ± 0.005, pre-E 1.08 ± 0.03; and phospho-eNOS: control 0.89 ± 0.28; pre-E 0.892 ± 0.32.

### 3.4. Ratios of eNOS to Phospho-eNOS and eNOS to Hsp90 Are Not Changed in Late Onset Pre-E

Immunoprecipitations were performed to gain insight into the association of eNOS with phospho-eNOS, and the association is unchanged indicating that eNOS is not synthesizing O_2_^−^ (Figures 3(b) and 3(c)) (control: 1.02 ± 0.05; pre-E: 0.96 ± 0.05, panel (b)) and eNOS and HSP90 (control: 1.12 ± 0.07; pre-E: 1.04 ± 0.09, panel (c)).

### 3.5. Red Light Treatment Enhances the NO Levels in Trophoblasts

**Figure 4:**
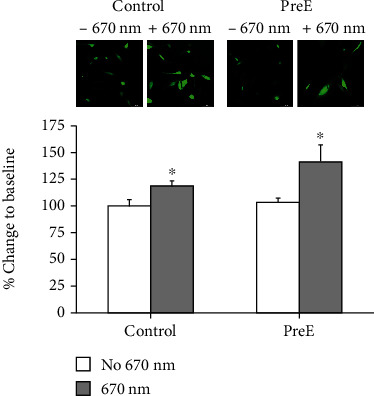
Red light treatment enhances the NO levels in trophoblasts. NO levels in trophoblast cells on control and pre-E matrices with DAF fluorescence. 670 nm light treatment resulted in a more extensive increase in the case of pre-E vs control (*p* ≤ 0.05, *n* = 4).

We also examined the NO levels in trophoblast cells on control and pre-E matrices with DAF fluorescence. Although the native NO levels were not statistically different, 670 nm light treatment resulted in a more extensive increase in the case of pre-E vs control (Figure 4).

### 3.6. pECM Is Oxidized in Late Onset Pre-E

**Figure 5:**
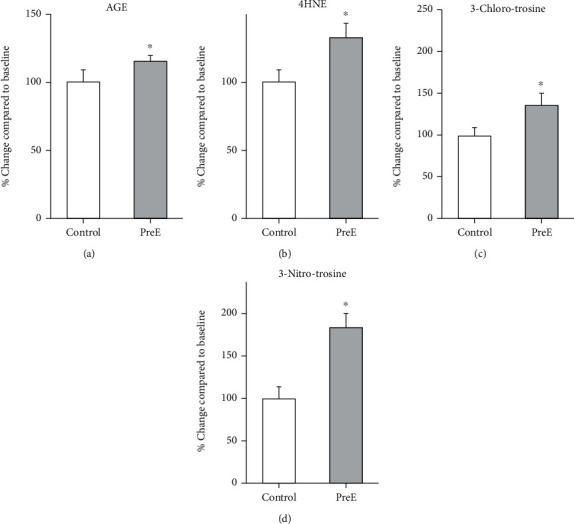
These oxidative adducts were increased in the pre-E placental sample. (a) AGE is significantly increased in late onset pre-E, (b) 4-HNE is significantly increased in late onset pre-E; (c) 3-chlorotyrosine was significantly increased in late onset pre-E; and (d) 3-Nitrotyrosine is significantly increased in late onset pre-E. (*p* ≤ 0.05, *n* = 4).

To determine the oxidative adducts on pECM as a readout for oxidation in late onset, AGE and lipid peroxidation end product 4HNE were measured by western blot analysis in placentas from healthy patients and patients diagnosed with late onset pre-E (Figure S1). These oxidative adducts were increased in the pre-E placental sample (Figures 5(a) and 5(b)) (AGE control: 100 ± 9.27, pre-E: 128.95 ± 6.93, 4HNE control: 100 ± 4.27, pre-E: 131.99 ± 10.08). Pre-E cellular microenvironment led to an increase of 3-ClTyr and 3-NTyr oxidative adducts when compared to control placental ECM (Figures 5(c) and 5(d)) (3-ClTyr: control 100 ± 10.28; pre-E 135 ± 14.5, 3-NTyr: control 100 ± 12.98; pre-E 182 ± 21.14).

### 3.7. Migration of Extravillous Trophoblasts Cells on Preeclamptic Matrix

**Figure 6:**
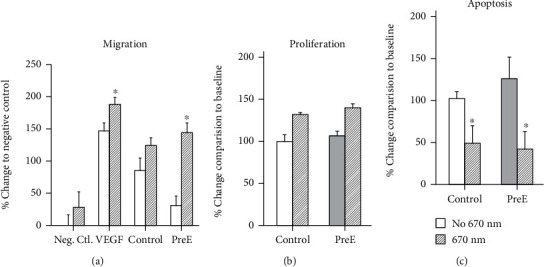
(a) 670 nm light improves migration of trophoblasts on pre-E pECM. (b) 670 nm light improves proliferation of trophoblasts on pre-E pECM. (c) 670 nm light reduces the number of apoptotic trophoblasts on pre-E pECM. (*p* ≤ 0.05, *n* = 4).

Trophoblast migration is of great importance for the embedding of trophoblasts into the maternal uterine vessels. The migration of HTR8/SVneo cells is reduced when they are cultured on fibrin gel blended with extracellular matrix derived from patients suffering from preeclampsia compared to fibrin gel blended with pECM from healthy patients (Figure 6(a)) (control: 86.29 ± 18.59; pre-E: 30.16 ± 15.49). 670 nm light treatment enhanced the migration of HTR8/SVneo cells cultured on control and pre-E pECM (control: 125 ± 11.97; pre-E: 144.83 ± 16.01). Negative control was 0 ± 16.47 without 670 nm, with 670 nm 27.74 ± 25.02, while VEGF was used as positive control without 670 nm 148.22 ± 12.86, without 670 nm 188.87 ± 12.03.

### 3.8. Extravillous Trophoblast Proliferation Is Unchanged when Cultured on Preeclamptic Matrix

Proliferation of trophoblasts contributes to the crucial embedding of trophoblasts in the uterine vessels. HTR8/SVneo cell proliferation is not enhanced when cultured on pECM from patients suffering from preeclampsia compared to pECM from healthy patients (Figure 6(b)) (control: 100% ± 7.04; pre-E: 108% ± 4.33). 670 nm light treatment enhanced proliferation of HTR8/SVneo cells both on control and on placental ECMs (control: 133% ± 1.67; pre-E: 141% ± 3.92).

### 3.9. Extravillous Trophoblast Apoptosis Is Increased on Pre-E Placental ECM

Apoptosis was determined based on the importance of the balance between proliferation and apoptosis [12]. HTR8/SVneo apoptosis was significantly increased when cultured on preeclamptic placental ECM compared to apoptosis rate of HTR8/SVneo on healthy control placental ECM (Figure 6(c)) (control:100% ± 10.2, pre-E:125% ± 18.6). 670 nm light treatment decreased the number of apoptotic HTR8/SVneo cells significantly (control: 50% ± 24.2, pre-E: 42% ± 21.2).

### 3.10. 670 nm Light Increases Expression Level of Placental Growth Factor (PLGF) and Transforming Growth Factor (TGF*β*) in HTR8/SVneo Cells when Cultured on Preeclamptic Matrix

**Figure 7:**
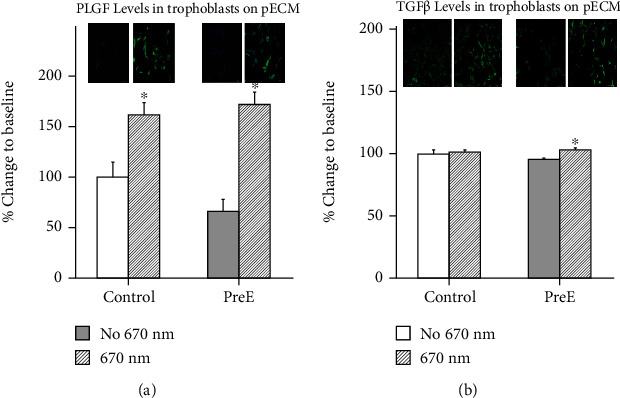
(a) 670 nm light increases expression level of Placental Growth Factor (PLGF) and (b) Transforming Growth Factor (TGF*β*) in HTR8/SVneo cells when cultured on preeclamptic matrix; (a) PLGF expression. (b) TGF*β* expression (*p* ≤ 0.05, *n* = 4).

Significantly lower PLGF levels were measured on pECM from pre-E patients when compared to healthy subjects. 670 nm light treatment significantly increased the expression level of PLGF in HTR8/SVneo cells cultured on pECM from healthy patients and patients diagnosed with late onset preeclampsia (light: control 162 ± 12.07; pre-E 172 ± 12.22) compared to untreated cells (no light: control 100 ± 15.8; pre-E 67 ± 9.5) in both groups (Figure 7(a)). TGF*β* expression levels were significantly decreased in HTR8/SVneo cells cultured on placental ECM from patients diagnosed with late onset preeclampsia compared to HTR8/SVneo cultured on pECM from healthy patients (no light: control 100 ± 3.05; pre-E 95 ± 0.88). 670 nm light treatment restored the expression level of TGF*β* in HTR8/SVneo cultured on pECM from patients diagnosed with late onset preeclampsia compared to HTR8/SVneo cultured on pECM from healthy patients (light: control 101 ± 1.77; pre-E 103 ± 1.12). 670 nm light treatment did not significantly increase the expression level of TGF*β* in HTR8/SVneo cultured on pECM from healthy patients (Figure 7(b)).

## 4. Discussion

We showed that (i) the ECM is oxidized in placentas derived from patients with a late onset preeclampsia diagnosis which is further confirmed by the detection of changes of expression of two major oxidation markers 3-chlorotyrosine and 3-nitrotyrosine, (ii) this oxidized environment led to change of trophoblast proliferation, migration, and apoptosis, as well as a decrease of Placental Growth Factor and Transforming Growth Factor *β* expression, (iii) an application of 670 nm light reversed the permutations of late onset pre-E. Our data demonstrate that the ECM from placenta and, hence, the cellular microenvironment have great impact on trophoblast signaling. Late onset pre-E pECM alters the trophoblast behavior, and photobiomodulation with 670 nm light restored the trophoblast behavior to a behavior found in trophoblasts cultured on healthy pECM.

Photobiomodulation is a novel, noninvasive modality [13] to increase NO availability [13, 14] in a disease state that is known for low bioavailability of NO. In our hands, 670 nm light increased the NO levels in trophoblasts and improved trophoblast behavior. Photobiomodulation is broadly studied as phototherapy in various diseases due to its low side effect profile [15]. The most extensively studied spectrum is 630 to 830 nm range because of its ability to penetrate with no heat production [16]. Even LED saunas with this light spectrum are commercially available. Previous data from our lab demonstrate induction of angiogenesis and collateralization in various disease states like diabetes and fibrosis with 670 nm light [2, 3, 6]. We were also able to show the cardioprotective effect of 670 nm light treatment [13]. Besides our success using 670 nm light in laboratory settings, 670 nm light is used in clinical application, for example, for wound healing, retina healing, healing of episiotomies, treatment of perennial allergic rhinitis, and relief of back pain [17–21].

The application of energy is unspecific and hence a caveat in our study. This unspecificity is reflected in some of our data resulting in a light effect in control and PE groups especially visible in the migration and proliferation assays.

Placental extracellular matrix is altered in preeclampsia. Our findings show a change in the expression of collagen-I, galectin-3, and heparan-sulfate proteoglycan 2. Collagens I, III, IV, and V are involved in the placentation process, and trophoblasts are synthesizing them leading to placental insufficiency [22–24]. The development of placental fibrosis in preeclampsia is a well-studied process [24, 25]. Collagen-I deposition is increased in fibrosis leading to preeclampsia [26]. Collagen-I expression was increased in our late-onset preeclampsia samples compared to our healthy placental samples suggesting an increase in fibrosis. Galectin-3's function is not well understood. Its increased expression is associated with preeclampsia, maternal morbidity, and mortality [27, 28]. In the preeclamptic ECM samples, we determined an increase in galectin-3 expression in agreement with published data [29]. Heparan-sulfate proteoglycan 2, perlecan, a macromolecule expressed in the placenta, is crucial for cell regulatory processes [30] and involved in the development of inflammation and fibrosis. Chui et al. describe the reduction of heparan-sulfate proteoglycan 2 expression in preeclampsia [31] which confirms our similar findings. A common mantra in the literature is an increase of oxidative stress as a cause for preeclampsia [5]. Many women diagnosed with preeclampsia have already underlying hypertension, a sign of increased oxidative stress. The persistent oxidative stress hampers the remodeling of spiral arteries resulting in a dysfunctional placenta which leads to a hypoxic environment and consequently to further oxidative stress [32, 33]. We do show an increase in the expression of oxidative adducts like AGE and 4HNE as well as chlorotyrosine and nitrotyrosine at that time point suggesting an ongoing inflammatory process. Oxidative adducts have been described in the literature to be elevated in preeclampsia [34–38].

Our findings demonstrate that in late onset preeclampsia, the reduction in the activity of eNOS in preeclamptic placentas compared to healthy placentas is not taking place. eNOS is coupled to Hsp90, and NO production is not hindered. Hence, we conclude that the availability of NO is not reduced, as it is described in the literature for early onset preeclampsia [5, 9, 39]. Moreover, 670 nm light treatment triggers enhanced NO availability most probably from additional internal NO sources (3, 14).

PLGF and TGF*β* are two growth factors which are important for migration and proliferation of extravillous trophoblasts. Our data show that migration of extravillous trophoblasts cultured in the preeclamptic ECM is impaired. We also found that PLGF expression is decreased in our preeclamptic samples. This decrease is closely linked in the signaling cascade with TGF*β* [40, 41]. The reduction in PLGF resulted in an increase of sFlt, the VEGF1 receptor highlighting the reciprocal relationship of these two signaling proteins [42]. Low levels of PLGF in the bloodstream is an indicator for the development of preeclampsia. Karumanchi suggested PLGF as a potential treatment for preeclampsia. PLGF binds to sFlt, the soluble splice variant of the VEGFR1 receptor. sFlt binds PLGF competitively; therefore, PLGF cannot exhibit its proper function [43]. Phototherapy improved the expression of these important players and consequentially improved cellular health.

Additional evidence suggests the altered placental microenvironment affect trophoblast behavior, migration, proliferation, and apoptosis. In late onset pre-E, the invading trophoblasts do not penetrate the spiral arteries leading to decreased blood vessel dilation and ischemia [44]. Peng et al. described that the penetration depth of trophoblasts was reduced in pre-E changing the vessel remodeling [45]. Our findings suggest that trophoblast migration is reduced, proliferation is intact, and apoptosis is increased when cultured on late onset pre-E pECM. These findings contrast with the published data which show overwhelmingly an increased number of apoptotic cells and decreased proliferation [46–48]. At this point, we do not have an explanation for this finding and further studies are being conducted. Furthermore, our eNOS, Hsp90 data is only trending. Our working hypothesis currently is that this is probably caused by the focus on late onset preeclampsia with a milder disease progression. Studies are being conducted on early onset preeclampsia samples to address these unanswered questions.

Based on our data, we conclude that oxidative processes increase oxidative adducts on pECM and alter the pECM components resulting in a deteriorating microenvironment. We showed that 670 nm light administration mitigated the microenvironment and consequently the trophoblast behavior in the late onset preeclampsia environment.

## Data Availability

Collected data can be requested from the corresponding author upon inquiry.

## Supplementary Materials

Supplementary Materials One supplemental figure is added to support our findings. Figure S1 shows representative western images supporting Figure 5.
